# Open versus arthroscopic Latarjet procedures for the treatment of shoulder instability: a systematic review of comparative studies

**DOI:** 10.1186/s12891-018-2188-2

**Published:** 2018-07-25

**Authors:** Nolan S. Horner, Paul A. Moroz, Raman Bhullar, Anthony Habib, Nicole Simunovic, Ivan Wong, Asheesh Bedi, Olufemi R. Ayeni

**Affiliations:** 1 Division of Orthopaedic Surgery, Department of Surgery, McMaster University, 1200 Main St W, Room 4E15, Hamilton, ON L8N 3Z5 Canada; 2 Faculty of Medicine, University of British Columbia, Vancouver, BC Canada; 3 Faculty of Medicine, Royal College of Surgeons in Ireland - Medical University of Bahrain, Manama, Bahrain; 4 Department of Health Research Methods, Evidence and Impact, McMaster University, Hamilton, ON Canada; 5 Department of Orthopaedic Surgery, Dalhousie University and Nova Scotia Health Authority, Halifax, NS Canada; 6 MedSport, Department of Orthopaedic Surgery, University of Michigan, Ann Arbor, MI USA

**Keywords:** Latarjet, Shoulder instability, Arthroscopy, Bone block, Instability, Dislocation

## Abstract

**Background:**

The arthroscopic and open Latarjet procedures are both known to successfully treat shoulder instability with high success rates. The objective of this study was to compare the clinical outcomes and positioning of the coracoid graft and screws between the arthroscopic and open Latarjet procedures.

**Methods:**

The electronic databases MEDLINE, EMBASE, and PubMed were searched for relevant studies between database creation and 2018. Only studies directly comparing open and arthroscopic Latarjet procedures were included.

**Results:**

There were 8 included studies, with a total of 580 patients treated arthroscopically and 362 patients treated with an open Latarjet procedure. Several papers found significantly better standardized outcome scores for either the open or arthroscopic procedure but these findings were not consistent across papers. Patients treated with arthroscopic Latarjet procedures had significantly lower initial post-operative pain, however pain scores became equivalent by one month post-operatively. Three of the five included studies found no significant difference in the coracoid graft positioning and two of three included studies found no significant difference in screw divergence angles between the two techniques. Arthroscopic procedures (112.2 min) appear to take, on average, longer than open procedures (93.3 min). However, operative times and complication rates decrease with surgeon experience with the arthroscopic procedure. Overall 3.8% of the patients treated arthroscopically and 6.4% of the patients treated with the open procedure went on to have post-operative complications.

**Conclusions:**

Both open and arthroscopic Latarjet procedures can be used to effectively treat shoulder instability with similarly low rates of complications, recurrent instability and need for revision surgery. Arthroscopic Latarjet procedures are associated with less early post-operative pain but require increased operative time. The evidence does not support there being any significant difference in graft or screw positioning between the two techniques. At this time neither procedure shows clear superiority over the other.

## Background

The shoulder is the most commonly dislocated joint and most frequently dislocates anteriorly. The Latarjet is a commonly performed procedure in the treatment of recurrent anterior shoulder instability. This procedure was first characterized in 1954 and modified multiple times since its conception [1]. This procedure classically involves a deltopectoral approach in order to transfer the coracoid process, along with attached soft tissue to the anterior-inferior border of the glenoid. This stabilizes the shoulder through a triple mechanism which uses the conjoint tendon as a sling and the coracoid process as a bony block, while repairing the capsule via fixation to the coracoacromial ligament [2]. That being said, there still exists a number of controversies surrounding the optimal orientation, size and positioning of the graft when preforming the Latarjet procedure [3–5]. For instance, one study found that the Latarjet procedure which involves transfer of the entire horizontal pillar of the coracoid better restored stiffness to the glenohumeral joint in comparison to the Bristow procedure where only the tip of the coracoid is transferred [5]. The Latarjet is a well-established treatment option with good evidence for favourable long term outcomes [6]. Re-dislocation rates following a successful Latarjet procedure are estimated to be 4 to 5% [7].

Advances in technology have recently made an arthroscopic approach to the Latarjet procedure a possibility [8]. Lafosse et al. has proposed that the arthroscopic approach offers advantages such as more accurate bone graft placement, quicker functional recovery, decreased stiffness, and cosmetic benefits [9]. Despite minimal cases of recurrent dislocation in both surgical approaches, theorized disadvantages of the arthroscopic Latarjet include increased cost, longer surgery time, and increased complication rates stemming from challenging graft fixation [10, 11]. This may partially be explained by the arthroscopic Latarjet’s complexity and learning curve. Several studies have described a more prolonged learning curve in the arthroscopic Latarjet procedure [10]. However, there currently exist no consensus on whether the arthroscopic or open Latarjet procedure offers overall superior outcomes and/or complication rates.

The purpose of this study is to compare the standardized clinical outcome scores, rates of complication, accuracy of graft and screw positioning and rates of recurrent dislocation between the open and arthroscopic Latarjet procedures by systematically reviewing the literature for comparative studies.

## Methods

### Search strategy

**Fig. 1:**
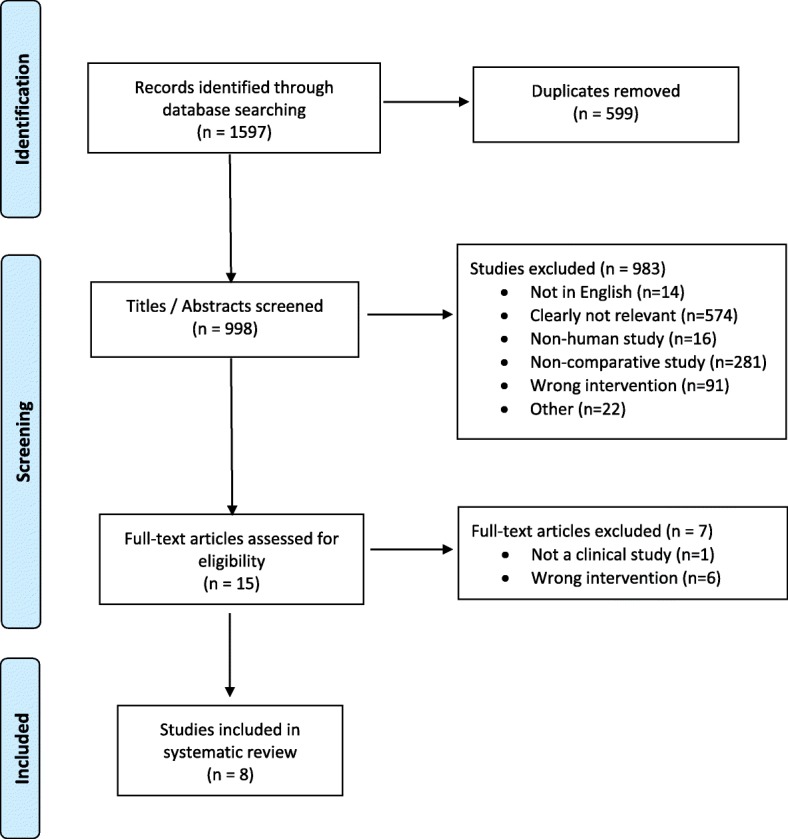
Outline of systematic search strategy used

Three online databases (EMBASE, MEDLINE, and PubMed) were searched by two reviewers (P.M., R.B.) for literature comparing any clinical outcomes or positioning of the graft or screws after arthroscopic and open Latarjet procedures in male and female patients of all ages for the treatment of shoulder instability (Fig. 1). The database search was conducted on March 1, 2018. The inclusion criteria for this search was: (1) Studies comparing outcomes and/or failure rates between open and arthroscopic Latarjet procedures for anterior shoulder instability; (2) Studies comparing the accuracy of the coracoid bone graft or screw positioning; (3) male and female patients of all ages; (4) studies published in English; (5) studies on humans. The exclusion criteria were: (1) any non-surgical treatment studies (e.g. technique articles without outcomes, cadaver studies, review articles, etc.); (2) non-comparative studies.

The following search terms were used: “Latarjet”, “Bristow”, “Latarjet-Bristow”, “Latarjet-patte”, “Coracoid” and “Bone block”, and “Coracoid” and “Transfer”. Both key term and subject heading search methods were used where applicable. A detailed search strategy is presented in Appendix 1: Table 4.

### Study screening

Two reviewers (P.M., R.B.) independently screened the titles of the retrieved papers. Any included studies were then screened by abstract. Disagreements at either of these screening stages were resolved by including the papers for full text review. Any disagreements at the full text screening stage were discussed by the reviewers and resolved by a third reviewer (N.H.). A list of references for the papers deemed ineligible at the full text review stage can be found in Appendix 2.

### Quality assessment of included studies

A quality assessment of the included studies was completed using the Methodological Index for Non-Randomized Studies (MINORS) Criteria [12]. MINORS is a validated scoring tool for non-randomized studies (e.g. case reports, case series, cohort studies etc.). Each of the 12 items in the MINORS criteria is given a score of 0, 1 or 2 - giving a maximum score of 24 for comparative studies. To the author’s knowledge, there is no evidence to categorize the MINORS score. Thus, the MINORS score was categorized apriori as follows: 0–6 to indicate very low quality evidence; 7–10 to indicate low quality of evidence; 10–14 to indicate fair quality of evidence; and > 16 to indicate a good quality of evidence for non-randomized studies.

### Data abstraction

Two reviewers (NH, RB) independently abstracted relevant study data from the final pool of included articles and recorded this data in a Microsoft Excel (2013) spreadsheet designed a priori. Demographic information included author, year of publication, sample size, study design, level of evidence, patient demographics (i.e. sex, age, affected shoulder, follow-up time, % lost to follow-up, etc.) and details of surgery. In addition to demographic information any clinical or information regarding graft or screw positioning was documented. The number of patients requiring further surgery was also abstracted. Finally, any minor or major complications associated with procedures were recorded.

### Statistical analysis

Interobserver agreement was assessed at each stage of study screening by calculating a weighted k (kappa). The agreement between the two reviewers assessing the MINORS score in duplicate across all studies was calculated using an intraclass correlation coefficient (ICC), which evaluates the consistency of multiple observers measuring the same groups of data. Agreement was categorized a priori as follows: k > 0.61 to indicate substantial agreement, 0.21 < k < 0.60 to indicate moderate agreement, and k < 0.20 to indicate slight agreement. Statistics describing the data collected from the included papers was presented as means, ranges, and measures of variance where appropriate. A meta-analysis was not performed in this study due to the high heterogeneity amongst reported outcomes and the specifics of how the procedure was done.

## Results

### Study identification and characteristics

Our initial literature search yielded 1597 studies, of which 8 met the inclusion and exclusion criteria for this review (Fig. 1). There was excellent agreement among reviewers at the title (k = 0.76; 95% CI, 0.71 to 0.80), abstract (k = 0.94; 95% CI, 0.90 to 0.98.) and full-text screening (k = 1.0; 95% CI, 1.0 to 1.0) stages. All included studies were published between 2016 and 2018. This included a total of 942 patients, including 580 patients treated arthroscopically and 362 patients treated with an open Latarjet procedure. The mean sample size of the included studies was 117.8 (range 46–390) patients. 81.0% of the patients treated across the studies were male, with a mean age of 27.7 (range 13.6–66) years and mean follow-up 20.6 months. 36.5% of the patients were reported as being lost to follow-up.

Only two studies commented on whether the Latarjet procedures were done as a primary surgery or as a revision surgery after previous shoulder stabilization surgery. Amongst these two studies 98.0% (*n* = 100) of the patients treated arthroscopically and 98.9% (*n* = 94) of the patients treated with an open procedure had not had previous shoulder stabilization surgery. Study demographics are presented in Table 1.

**Table 1:** Characteristics of included studies

Primary Author, Year	Location	Study Design	Level of Evidence	Inclusion Criteria	Exclusion Criteria	Sample Size – Patients	% Male	Mean age (years)	Follow-up [months]	% Lost to Follow-Up	MINORs score
Cunningham G,2016 [10]	Switzerland	Retrospective comparative cohort	III	Significant bone loss with indication for Latarjet procedure according to established criteria	Any patient < 16 years of age or with history of previous bone block procedure	Arthroscopic – 28 Open- 36	90.6%	26+/−7.6 (15–45)	6.6+/− 5.9 (1.5–36)	0%	19
Marion B, 2017 [13]	France	Prospective comparative cohort	II	Chronic post-traumatic anterior glenohumoral instability, based on instability severity index score > 3	Not specified	Arthroscopic – 36 Open – 22	77.6%	26.9+/− 7.7	29.8+/− 4.4	3.4%	18
Kordasiewicz B, 2017 [16]	Poland	Retrospective comparative cohort	III	All patients undergoing primary shoulder stabilization between 2006 and 2013	Revision cases	Arthroscopic – 62 Open - 48	Not specified	26.9	35.1	8.2%	18
Kordasiewicz B, 2018 [19]	Poland	Retrospective comparative cohort	III	All patients undergoing primary shoulder stabilization between 2006 and 2013	Revision cases	Arthroscopic – 62 Open - 48	Not specified	26.9	36.8	7.6%	18
Metais P, 2016 [15]	France	Prospective comparative cohort	II	Not specified	Not specified	Arthroscopic – 286 Open – 104	Not specified	27.8 (13.6–66.6)	22.7+/−4.1	63.6%	17
Nourissat G, 2016 [14]	France	Prospective comparative cohort	II	Older than 18 years old, experienced at least one shoulder dislocation episode and undergoing anterior bone block surgery	Patients with post-operative complications who required revision surgery	Arthroscopic – 99 Open – 85	Not specified	Not specified	5.5 (1–12)	46.2%	18
Russo A, 2017 [18]	Italy	Prospective comparative cohort	II	Shoulder stabilization with either open or arthroscopic latarjet	Age > 50 years, rotator cuff tears, multidirectional instability, systemic disorders.	Arthroscopic – 25 Open – 21	93.5%	Not specified	Minimum 1-year follow-up	0%	16
Zhu Y, 2017 [17]	China	Prospective comparative cohort	II	1. Diagnosis of recurrent anterior shoulder dislocation 2. Glenoid bone defect greater than 20% on CT 3. Treatment with open Latarjet or arthroscopic Latarjet 4. Agreement to participate in the study 5. Post-op follow up more than 2 years	1. Concomitant musculoskeletal injuries or neurovascular disorders of the ipsilateral shoulder 2. Multi- directional shoulder instability 3. Uncontrolled epilepsy or psychological condition that prevented patients from following post-op rehab	Arthroscopic – 44 Open – 46	75% (68)	30.1	28.8	0%	19

### Study quality

There was a total of three (37.5%) level III and five (62.5%) level II studies that met the inclusion criteria (Table 1). The included studies had a mean MINORS score of 17.9 ± 1.0 which indicates a good quality of evidence amongst non-randomized studies (Table 1). There was high agreement (ICC = 0.96 (95% CI, 0.94 to 0.98)) amongst quality assessment scores of included studies using the prespecified criteria.

### Outcomes

A summary of the clinical outcomes scores presented in the include studies is presented in Table 2. A variety of scores were used including the visual analog scale (VAS), Western Ontario Shoulder Instability Index (WOSI), Rowe, Walch-Duplay, American Shoulder and Elbow Surgeons Shoulder Score (ASES) and Constant-Murley scores. Both studies that looked at early (< 1 month) post-operative pain found significantly less pain in the arthroscopic group [13, 14]. However one study found no difference in VAS pain scores once patients had reached 30 days post-operatively [14]. One study (*n* = 286 arthroscopic, 104 open, *p* < 0.05) found significantly better post-operative Walch-Duplay scores in the arthroscopic group [15], whereas the other two studies (n = 28 arthroscopic, 36 open, *p* > 0.05; *n* = 62 arthroscopic, 48 open, p > 0.05) reporting post-operative Walch-Duplay scores did not [10, 16]. Interestingly one study (*n* = 99 arthroscopic, 85 open, *p* < 0.05) reported significantly better post-operative WOSI scores in the arthroscopic group [14], whereas another study (*n* = 36 arthroscopic, 22 open, *p* = 0.03) found significantly better WOSI scores in the open group [13]. Similarly, one study (n = 62 arthroscopic, 48 open, p < 0.05) found significantly better Rowe scores in the open group [16], one study (*n* = 286 arthroscopic, 104 open, p < 0.05) found better Rowe scores in the arthroscopic group [14], and one study (*n* = 44 arthroscopic, 46 open, *p* = 0.181) found no significant difference [17]. No studies found significant differences in ASES or Constant-Murley scores.

**Table 2:** Clinical outcome scores reported in the included studies

Study	Arthroscopic Outcome	Open Outcomes	Significance
Cunningham G, 2016 [10]	Walch Duplay score − 88. Persistant apprehension - 4(5.5%)	Walch Duplay score − 91. Persistant apprehension - 0(0.0%)	Walch Duplay score - no significant difference
Marion B, 2017 [13]	VAS (1 week) - 1.2 +/−1.4 WOSI (2 years) - 372.1+/− 140.9	VAS (1 week) - 2.5+/− 1.4 WOSI (2 years) - 451+/− 158.7	VAS *p* = .002 WOSI *p* = 0.03
Kordasiewicz B, 2017 [16]	Walch - Duplay score - 76.7 Rowe - 78.9 VAS- 1.38 Satisfaction% - 91.9 Residual subjective apprehension - 31%	Walch - Duplay score - 83.9 Rowe - 87.8 VAS- 0.77 Satisfaction% - 96.8 Residual subjective apprehension 28.7%	p < 0.05 for Rowe and Subjective apprehension only. The rest did not reach statistical significance.
Metais P, 2016 [15]	Walch - Duplay score – 92.8. Rowe − 93.4	Walch - Duplay score - 85.9. Rowe − 83.9	Walch-Duplay score *p* < 0.0001 Rowe p < 0.0001
Nourissat G, 2016 [14]	VAS (30 days) - 1.2	VAS (30 days) - 1.6	VAS (30 days) *p* = 0.14 (note significantly lower VAS scores were found in arthroscopic group at earlier follow-ups). WOSI (6 months) - open had significantly better symptoms and sports/recreation/work scores than the arthroscopy group. No significant difference in Lifestyle and Emotion scores.
Zhu Y, 2017 [17]	ASES- 93.3+/− 9.9 Constant Murley Socre - 96.5+/− 3.8 Rowe - 97.1+/− 2.5	ASES- 93.0+/− 5.0 Constant Murley Socre - 95.0+/− 4.1 Rowe - 95.4+/− 5.0	ASES *p* = .917 Constant-Murley *p* = .223 Rowe *p* = .181

Five of the included studies reported on radiographic outcomes. Three of the five studies (*n* = 126 arthroscopic, 106 open, *p* > 0.05) found no significant difference in the coracoid graft positioning between the two techniques [13, 18, 19], one study (*n* = 25 arthroscopic, 21 open, *p* = 0.025) found the arthroscopic technique to be significantly more likely to have ideal graft positioning [18], and conversely one study (*n* = 44 arthroscopic, 46 open, *p* < 0.001) found the open procedure to be significantly more likely to have ideal graft positioning [17]. Three of the studies reported on screw divergence angles, two studies (*n* = 69 arthroscopic, 67 open, *p* = 0.10–0.12) found no significant difference between the two techniques [17, 18], and one study (*n* = 28 arthroscopic, 36 open, *p* = 0.017) found the open technique to have significantly less rates of excessively (> 10°) divergent screws [10].

The rates of re-operation, complications, and recurrent instability are shown in Table 3. Overall 3.8% of the patients treated arthroscopically and 6.4% of the patients treated open went on to have post-operative complications. The most common post-operative complications included recurrent instability (arthroscopic – 1.9%, open – 1.4%), graft fracture, failure or non-union (arthroscopic – 1.2%, open – 1.6%) and infection (arthroscopic – 0.9%, open – 1.1%) in both groups. A total of 4.1% of the arthroscopic patients and 3.0% of the open patients required revision surgery. Screw removal (arthroscopic – 1.1%, open – 0.8%) and need for revision due to recurrent instability (arthroscopic – 2.0%, open – 1.4%) were the two most common reasons for revision surgery in both groups. A total of six (1.1%) of the arthroscopic procedures had to be converted to open procedures due to technical difficulties. The average operative time for open procedure was 93.3 min and 112.2 min for arthroscopic procedures. In total 6 (1.1%) patients treated arthroscopically and 7 (2.0%) treated with an open procedure experienced intra-operative complications.

**Table 3:** Complications and reoperation rates reported amongst included studies

Study	Procedure	Number of intra-op complications	Number of post-op complications (including instability)	Number of Revision surgeries	Number of recurrent instability
Cunningham G, 2016 [10]	Open	0%	11%	0%	0%
	Arthroscopic	0%	29%	4%	4%
Marion B, 2017 [13]	Open	0%	0%	0%	0%
	Arthroscopic	0%	6%	8%	3%
Kordasiewicz B, 2017 [16]	Open	13%	13%	8%	6%
	Arthroscopic	8%	10%	13%	5%
Kordasiewicz B, 2018^a^ [19]	Open^a^	N/A	N/A	N/A	N/A
	Arthroscopic^a^	N/A	N/A	N/A	N/A
Metais P, 2016 [15]	Open	1%	13%	7%	2%
	Arthroscopic	0%	2%	4%	2%
Nourissat G, 2016 [14]	Open	0%	0%	0%	0%
	Arthroscopic	0%	0%	0%	0%
Russo A, 2017 [18]	Open	N/A	N/A	N/A	N/A
	Arthroscopic	N/A	N/A	N/A	N/A
Zhu Y, 2017 [17]	Open	0%	0%	0%	0%
	Arthroscopic	0%	0%	0%	0%

^a^Kordasiewicz B, 2018 only included hardware complications reported and overlapping patient populations with Kordasiewicz B, 2017, therefore Korasiewicz B, 2018 was not included in any complication calculations

One study which also looked at the learning curve associated with the arthroscopic Latarjet procedure also noted that operative time, rates of complications and need for conversion from arthroscopic to an open procedure due to technically difficulties decreased as surgeons gained experience with the procedure [10].

## Discussion

The results of the current study suggest that there is no clear superiority of the open versus arthroscopic approach for Latarjet procedures based on differences in complication rate or recurrence of instability. Several of the papers found superiority for individual standardized outcome scores for either the open or arthroscopic procedure, but these findings were not consistent across papers. Patients being treated with arthroscopic Latarjet procedures have lower reported pain scores in the first couple weeks post-operatively however these scores become equivalent to the open procedures by one month. The average time for the procedure was longer for the arthroscopic procedure compared to the open procedure however no statistical analysis to determine if this was significant was possible due to error of measurements not being reported within the studies. However, the studies did note a significant drop in operative time with the arthroscopic procedure as surgeon experience increased [10]. It should be noted that the studies did not comment on the amount of surgeon experience with each procedure prior to the initiation of the studies, which may have affected results if surgeons were more experienced with one procedure over the other.

Interestingly, the arthroscopic latarjet technique did not show improved positioning of the bone block or of the screws despite the theoretically improved visualization when placing the graft. This is of key importance given the known importance of positioning of the coracoid graft and resulting biomechanical stability of the shoulder [20]. Furthermore, screws that are divergent more than 10 degrees are known to put the suprascapular nerve at risk for injury [21].

We also found that both the arthroscopic and open Latarjet procedures both had relatively low and similar rates of major post-operative complications, recurrent instability and need for revision surgery. Unfortunately, we are unable to comment on whether there was a statistically significant difference in these rates due to the low overall event rate and the low number of studies available on this topic making any meta-analysis underpowered and with large range of variance. It should be noted that not all included Latarjet procedures were primary procedures which may have affected the rate of recurrent instability as rates of failure of the Latarjet procedure may be higher when done as a part of a revision surgery.

Only 1% of the total arthroscopic procedures were converted to open procedures due to technical difficulties. This may, however, be underestimated as it is unclear if all of the studies were reporting this measure. One study did comment on the fact that all of their intra-op conversions to open procedures occurred in their first third of cases with no conversion to open procedures in the remainder of their cases [10]. This suggests that the rates of conversion to open procedures may be very low once surgeons have performed a sufficient number of arthroscopic Latarjet procedures. In fact, several papers in the literature have found performing the Latarjet procedure arthroscopically to have a prolonged learning curve [10, 22].

There exists one previous systematic review on this topic which included 6 of the 8 studies included in this systematic review [23]. The conclusions of Hurley et al. are consistent with the findings of our systematic review which is that both the open and arthroscopic procedures offer significant improvement in clinical outcomes with similar complication rates [23]. However, this systematic review by Hurley et al. does not include all the available literature on the topic [23]. Furthermore, this study quantitatively synthesizes data from multiple studies through a meta-analysis even though multiple studies had retrospective design which generally increases heterogeneity and reduces precision of estimates in a meta-analysis.

This study has numerous strengths including the rigorous methodology which was used in this systematic review. Specifically, a broad search strategy spanning multiple databases was used to ensure that as much of the relevant literature was included as possible. The screening of studies was done in duplicate in order to limit reviewer bias.

The main limitation in this study was the quality of evidence available on the topic. Specifically, there currently exists no randomized studies comparing the arthroscopic and open Latarjet procedures. Furthermore, although only comparative studies were included, no meta-analysis was possible due to the significant heterogeneity in outcomes reported across the studies. Although the outcome measures used were generally appropriate and validated for this patient population, one study used the constant-Murley score as an outcome which previous authors have found to be a poor outcome measure for shoulder instability [24]. Additionally, the average follow-up of the included studies was less than 2 years and therefore differences in long-term outcomes after arthroscopic and open Latarjet procedures cannot be commented on. This is key as certain outcomes such as the development of osteoarthritis after the Latarjet procedure may only be measurable with longer follow-ups [25]. There was also procedural differences in the study such as screw versus endobutton fixation and open versus a mini-open approach. That being said, the literature on this subject is likely to improve as the arthroscopic Latarjet procedures have only recently been described, and in fact all the included studies in this systematic review were published as recently as 2016. Future large randomized studies comparing the arthroscopic and open procedures will provide further clarity on the possible superiority of one technique over the other as well as the specific indications for each procedure.

## Conclusions

Both open and arthroscopic Latarjet procedures can be used to effectively treat shoulder instability with similarly low rates of complications, recurrent instability and need for revision surgery. Arthroscopic Latarjet procedures are associated with less early post-operative pain but require increased operative time. The evidence does not support there being any significant difference in graft or screw positioning between the two techniques. At this time neither procedure shows clear superiority over the other.

## Appendix 1

**Table 4:** Search Strategy

	MEDLINE	EMBASE	PubMED
Search strategy	1. Latarjet.mp. 2. Bristow.mp. 3. Latarjet-bristow.mp. 4. Latarjet-patte.mp. 5. Coracoid.mp. 6. Bone Block.mp. 7. Transfer.mp. 8. 5 and 6 9. 5 and 7 10. 1 or 2 or 3 or 4 or 8 or 9	1. Latarjet.mp. 2. Bristow.mp. 3. Latarjet-bristow.mp. 4. Latarjet-patte.mp. 5 Coracoid.mp. 6. Bone Block.mp. 7. Transfer.mp. 8. 5 and 6 9. 5 and 7 10. 1 or 2 or 3 or 4 or 8 or 9	(Latarjet) OR (Bristow) OR (Latarjet-Bristow) OR (Latarjet-patte) or ((Coracoid) AND ((Bone Block) OR (transfer))
Number of papers retrieved	556	724	317

## Appendix 2

### References of Studies eliminated at Full Text Screen

1. Bessière C, Trojani C, Carles M, Mehta SS, Boileau P. The open latarjet procedure is more reliable in terms of shoulder stability than arthroscopic bankart repair. *Clin Orthop Relat Res* 2014;472(8):2345–2351. 10.1007/s11999-014-3550-9.

2. Bokshan SL, DeFroda SF, Owens BD. Comparison of 30-Day Morbidity and Mortality After Arthroscopic Bankart, Open Bankart, and Latarjet-Bristow Procedures: A Review of 2864 Cases. *Orthop J Sports Med* 2017;5(7):2325967117713163. 10.1177/2325967117713163.

3. Khan A, Samba A, Pereira B, Canavese F. Anterior dislocation of the shoulder in skeletally immature patients: comparison between non-operative treatment versus open Latarjet’s procedure. *Bone Joint J* 2014;96-B(3):354–359. 10.1302/0301-620X.96B3.32167.

4. Makhni EC, Lamba N, Swart E, et al. Revision Arthroscopic Repair Versus Latarjet Procedure in Patients With Recurrent Instability After Initial Repair Attempt: A Cost-Effectiveness Model. *Arthroscopy* 2016;32(9):1764–1770. 10.1016/j.arthro.2016.01.062.

5. Randelli P, Fossati C, Stoppani C, Evola FR, De Girolamo L. Open Latarjet versus arthroscopic Latarjet: clinical results and cost analysis. *Knee Surgery, Sport Traumatol Arthrosc* 2016;24(2):526–532. 10.1007/s00167-015-3978-9.

6. Zhang AL, Montgomery SR, Ngo SS, Hame SL, Wang JC, Gamradt SC. Arthroscopic versus open shoulder stabilization: current practice patterns in the United States. *Arthroscopy* 2014;30(4):436–443. 10.1016/j.arthro.2013.12.013.

6. Zimmermann SM, Scheyerer MJ, Farshad M, Catanzaro S, Rahm S, Gerber C. Long-Term Restoration of Anterior Shoulder Stability: A Retrospective Analysis of Arthroscopic Bankart Repair Versus Open Latarjet Procedure. *J Bone Joint Surg Am* 2016;98(23):1954–1961. 10.2106/JBJS.15.01398

## Abbreviations

ASES: American Shoulder and Elbow Surgeons Shoulder Score
ICC: Intraclass correlation coefficient
MINORS: Methodological Index for Non-Randomized Studies
VAS: Visual analog scale
WOSI: Western Ontario Shoulder Instability Index
